# Multiple Spatial and Climatic Conditions Affect Kingbird Flycatchers' Clutch and Egg Sizes

**DOI:** 10.1002/ece3.73528

**Published:** 2026-04-19

**Authors:** Marcelo Assis, Neander M. Heming, Miguel Â. Marini

**Affiliations:** ^1^ Programa de Pós‐Graduação Em Ecologia, Instituto de Ciências Biológicas Universidade de Brasília Brasília Distrito Federal Brazil; ^2^ Programa de Pós‐Graduação Em Zoologia, Instituto de Ciências Biológicas Universidade de Brasília Brasília Distrito Federal Brazil; ^3^ Programa de Pós‐Graduação Em Ecologia e Conservação da Biodiversidade, Departamento de Ciências Biológicas Universidade Estadual de Santa Cruz Ilhéus Bahia Brazil; ^4^ Departamento de Zoologia, Instituto de Ciências Biológicas Universidade de Brasília Brasília Distrito Federal Brazil

**Keywords:** biogeography, climate, clutch size, egg size, latitudinal effect, life‐history

## Abstract

Variation in avian clutch and egg sizes across geographical gradients has long been debated, particularly regarding the mechanisms underlying latitudinal clines in reproductive investment. These patterns are primarily driven by climatic conditions, with latitude acting as a proxy for environmental gradients. Here, we assembled a robust dataset of eggs and clutches of eight species of kingbird flycatchers (*Tyrannus*) covering a broad geographic range to test the hypothesis that clutch and egg size vary systematically along environmental gradients, and predict that egg and clutch size would (a) increase with latitude, (b) be larger in more seasonal climate categories, (c) be larger at sites with cooler winters, and (d) be larger at sites with warmer and wetter long‐term climatic conditions. We analysed clutch and egg data from 35 scientific egg collections representing the geographically widespread genus *Tyrannus*. After applying spatial, temporal and taxonomic data quality controls, we analysed the relationships between clutch and egg size and climatic variables. The analyses of 1332 clutches and 4680 eggs of eight species collected during 158 years (1858–2016) showed that clutch and egg sizes increased toward higher latitudes. Both traits varied according to major climate types, regional subclimates, and local temperature and precipitation conditions. More seasonal regions had the largest clutches, although sites with colder winters did not have the largest clutches. Egg size was larger at sites with less severe dry periods, and larger eggs were also associated with lower temperatures. Overall, residual variation in clutch and egg size is strongly associated with climatic conditions at both regional and local scales. These findings contribute to understanding how avian reproductive traits respond to climatic gradients and may help anticipate how species will respond to future climate scenarios.

## Graphical abstract

The study examined how clutch size and egg size in Tyrannus kingbirds vary across environmental gradients using a large dataset collected over 158 years. The results showed that these reproductive traits increase toward the poles and are strongly influenced by regional and local climatic conditions such as seasonality, temperature and precipitation. The findings suggest that climate‐adapted species may respond to interannual weather variations, providing important insights into how they might cope with future climate scenarios.

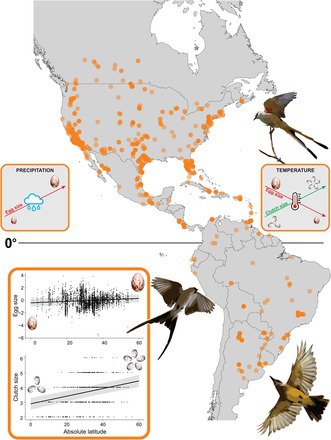

## 1 Introduction

Like other seasonally adapted organisms, birds' life‐history traits vary with environmental conditions, reflecting both plastic responses and adaptive differences among populations and species (Kuleska 1990; Forchhammer et al. 1998). Environment conditions act as drivers of organisms' physiology, and can help explain variations in breeding traits (Price and Liou 1989; Christians 2002; Bêty et al. 2003; Liu et al. 2018). Climate, and the inherent climatic seasonality (White and Hastings 2020), are key to bird phenology (Newton 1998). Few bird groups reproduce year‐round, even in tropical regions, where seasonality is often expressed through precipitation rather than temperature variation (Martin 1996; Stutchbury and Morton 2001). Migratory or resident birds must respond to climatic conditions at the breeding site (Ockendon et al. 2013).

Among the most evident clinal patterns in ecology is the variation in biological traits along latitudinal gradients (Hut et al. 2013). The relationship between clutch size and latitude is a well‐known pattern in avian reproductive investment, with larger clutches typically occurring at higher latitudes (Moreau 1944; Lack 1947; Ashmole 1963; Hõrak et al. 1995; Jetz et al. 2008; Stępniewski et al. 2021). A similar geographic pattern has also been described for animal body size (Stillwell 2010). However, large‐scale comparative analyses indicate that variation in clutch size is more strongly associated with ecological and life‐history characteristics than with body mass alone (Jetz et al. 2008). Several hypotheses have been proposed to explain latitudinal variation in avian clutch size, including food limitation and reproductive optimization (Lack 1947), constraints imposed by nest predation (Skutch 1949), and density‐dependent mechanisms linked to seasonal mortality (Ashmole 1963). Other explanations have also been proposed and reviewed in the literature (e.g., Lundblad and Conway 2021). Subsequent syntheses have further explored how ecological and life‐history factors interact to shape these patterns (Ricklefs 2000). Among these, one widely cited explanation is that increases in clutch size are related to the seasonality of resources (Ashmole 1963; Ricklefs 1980; Griebeler et al. 2004; Lundblad and Conway 2021). Latitude itself is therefore considered a proxy for environmental conditions that influence the phenological adaptation of species, such as seasonality (Lundblad and Conway 2021) and day length (Hut et al. 2013). Because seasonality can increase resource availability *per capita* during the breeding season, parents may allocate more energy to reproduction, allowing the production and maintenance of larger clutches.

Patterns and causes of egg size variation across geographic and environmental gradients remain poorly understood. Many ecologists argue that decreases in egg size may compensate for increases in clutch size (Smith and Fretwell 1974; Blackburn 1991; Christians 2002), suggesting that egg size may decrease with increasing latitude. However, empirical evidence for this trade‐off remains mixed, with some studies supporting this relationship (e.g., Pellerin et al. 2016), while others have found weak or no consistent association between egg size and clutch size across species and environments (e.g., Hõrak et al. 1995; Dolenec 2016). A positive relationship between egg and clutch size may indicate environments with sufficient resource availability to support increases in both reproductive investments (e.g., Hõrak et al. 1995; Dolenec 2016). Larger eggs may confer thermal advantages in colder environments because they lose heat more slowly than smaller eggs (Martin 2008). Moreover, incubation temperature can influence embryonic development and offspring phenotype, suggesting that thermal conditions may play an important role in shaping avian reproductive traits (DuRant et al. 2013). Although some studies have reported increases in egg size with latitude or across environmental gradients (Hõrak et al. 1995; Stępniewski et al. 2021) others found weak or no relationship between egg size and latitude (Guo and Lu 2022). Nevertheless, variation in egg size is correlated with female body size (Rahn et al. 1975; Bennett and Owens 2002; Martin et al. 2006) and incubation time (Rahn and Ar 1974). Because body size within related groups tends to increase with latitude (Blackburn et al. 1999; Stillwell 2010), latitudinal increases in adult body size may also be reflected in egg size through allometric relationships. Allometry explains about 80% of egg size variation in birds (Blackburn 1991). This relationship is highly conserved at higher taxonomic levels, such as orders, and may reflect phylogenetic constraints (Birchard and Deeming 2015). The remaining variation not explained by phylogenetic components is often influenced by ecological and environmental factors (Martin et al. 2006). Environmental conditions may therefore influence reproductive traits, although these responses are expected to occur within the constraints imposed by life‐history trade‐offs (Blackburn 1991).

Resource availability may be a key factor in fine‐tuning the timing of reproduction, as females schedule laying and hatching so that the period of greatest energetic demand by the offspring coincides with the expected peak of resources (Welty 1982; Dunn and Winkler 2008). Although studies have already diagnosed some of the residual variations in egg size (clutch size: Smith and Fretwell 1974; Blackburn 1991; Martin et al. 2006; Martin 2008; environmental conditions: Martin et al. 2006; Martin 2008; Heming and Marini 2015; migratory behaviour: Heming and Marini 2015; Sousa et al. 2024; and geographical variation: Martin et al. 2006), these variables still do not account for all the residual variation (Martin et al. 2006; Martin 2008). Hence, further investigation is needed to understand how reproductive traits vary within a group of birds over time and space (Heming and Marini 2015).

To investigate these patterns, we focused on kingbirds (*Tyrannus* spp.), a geographically widespread genus of tyrant flycatchers, and compiled an extensive dataset of clutch and egg size records from scientific egg collections spanning nearly the entire latitudinal distribution of the group across the Americas. Using this large‐scale dataset, we evaluated how clutch and egg size in kingbirds (*Tyrannus* spp.) varied in relation to geographic and climatic gradients. Specifically, we examined whether these reproductive traits vary according to (1) latitude, (2) major climate types based on the Köppen‐Geiger classification, (3) climate subtypes within these categories and (4) long‐term local climatic conditions derived from temperature and precipitation averages.

We tested the hypothesis that clutch and egg size vary systematically along environmental gradients. Based on existing hypotheses relating reproductive investment to climatic seasonality and geographic variation, we predict that these traits will: (a) increase with latitude, (b) be larger in more seasonal climates, (c) be larger at sites experiencing cooler winters and (d) be larger at sites experiencing warmer and wetter growing seasons (higher annual maximum temperatures and precipitation), which may reflect greater ecosystem productivity and resource availability during the breeding period.

## 2 Methods

### 2.1 Database

Information on clutch size and egg specimens of kingbird species (*Tyrannus* spp.) was obtained by examining scientific egg collections and photographing clutches in museum collections. Egg dimensions were later measured from the photographs using image analysis software. We chose the collections by researching and accessing the digitised and tabulated collections on their websites or scientific collection aggregators (e.g., GBIF, VertNet). With the knowledge on the contents of most part of these collections, we visited 35 museums in South America, the USA and Europe. We inspected the eggs to confirm that they matched the diagnostic characteristics of the family Tyrannidae. We excluded egg sets with eggs of abnormal colour, mark patterns or size for a given species. Then we checked the data shown on the respective labels/cards. With prior knowledge of the breeding biology of each species, we excluded clutches that did not accurately reflect the final clutch size, due to predation, parasitism or collecting bias. We only proceeded with those egg sets whose clutch size was reliable, even for the egg size analyses. In addition to clutch size, the records provided laying location and date, which was crucial to verify the data's integrity with information on each species natural history patterns, matching the location with species distributions (BirdLife International 2020) and the laying date with their recorded phenology. The first taxonomic classification whenever available (the original taxonomic identification recorded in the collections) was used to verify and update species names, accounting for synonymy and outdated taxonomic classifications. The taxonomic resolution for *Tyrannus* species followed the order: Sclater (1888), then Cory and Hellmayr (1927), Amadon et al. (1979) and Phillips (1994). Finally, we updated species names (and subspecies when possible) following eBird/Clements check‐list (Clements et al. 2025).

We took pictures of egg clutches arranged on a black base with a ruler scale and their respective collection labels/cards (Figure S1). We extracted egg dimensions from each picture using ImageJ software (Schneider et al. 2012) associated with the EggTools add‐on (Troscianko 2014). Besides the main dimensions (length, width, perimeter and area), the process allowed us to extract egg volume (mm^3^), which we treated here as ‘egg size’ (Figure S2). Establishing a maximum error of ±25 km, we used the clutch collection site present in the cards to obtain the geocode (decimal latitude and longitude) through the ggmap package, established through the centroid of the locality, defined by Google Maps service (Kahle and Wickham 2013). Every locality without a geocode but with provided localities with errors within a ±25 km limit was integrated into our database. This error was defined to be compatible with the respective climate information. With this level of coordinates resolution, we obtained climate conditions at the locality of egg‐laying.

**FIGURE 1:**
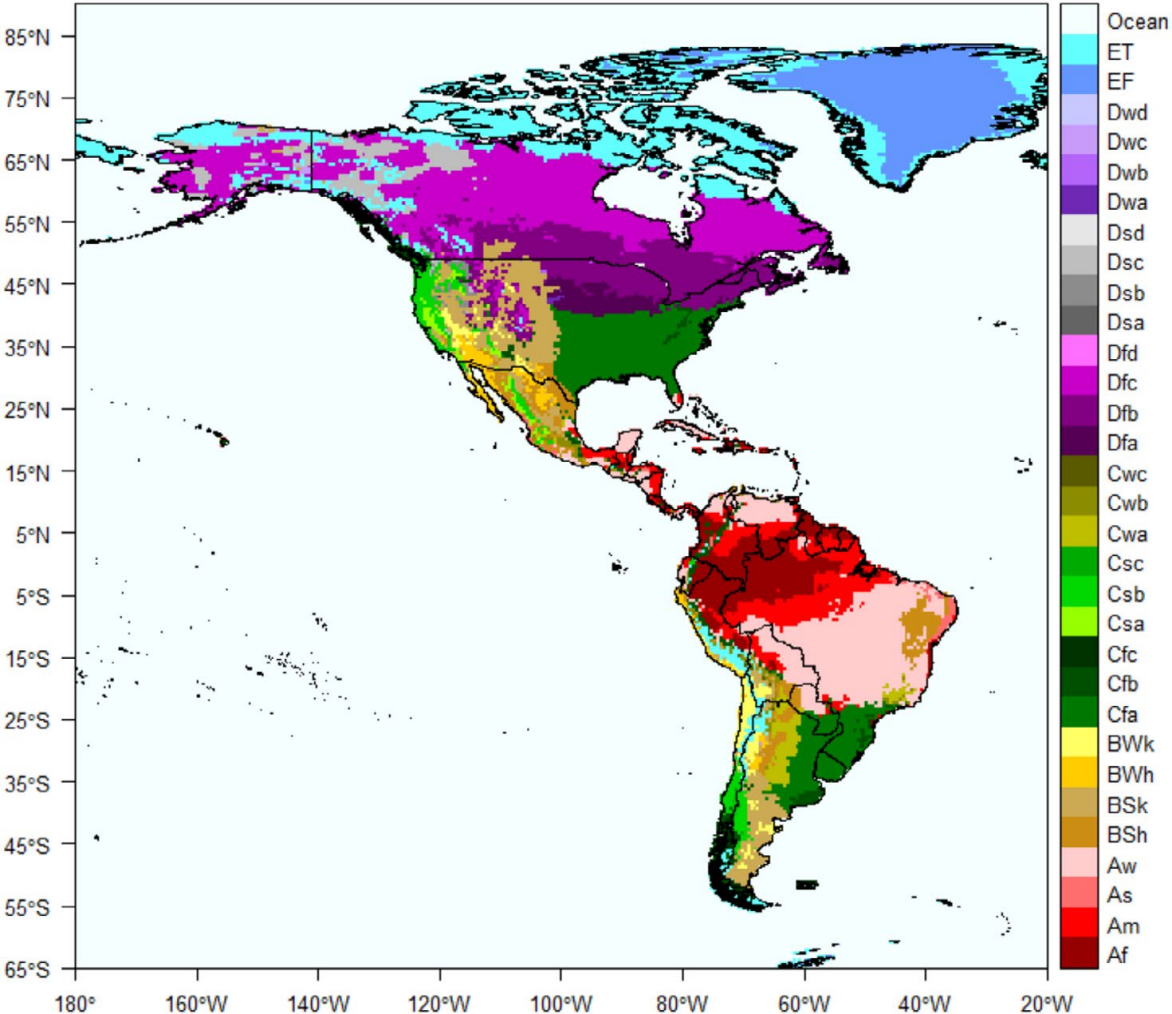
Map of the 31 Köppen‐Geiger subclimates of the Americas divided into five main climates (A: equatorial climates; B: arid climates; C: warm temperate climates; D: snow climates; and E: polar climates) generated from observed temperature and precipitation data from 25 years (1986–2010). *Source:* Adapted from http://koeppen‐geiger.vu‐wien.ac.at.

To classify the climate where clutches were collected, we used the Köppen‐Geiger geo‐climatic raster model (Kottek et al. 2006), assigning climate categories based on geographic coordinates of each clutch locality. Climate categories followed the Köppen‐Geiger classification system (Kottek et al. 2006), which defines five major climate types (A: equatorial; B: arid, C: warm temperate; D: snow climates; and E: polar) and their respective subtypes based on temperature and precipitation patterns. In total, this classification includes 31 climate categories (e.g., Af: equatorial rainforest; fully humid; Csa: warm temperate climate with dry and hot summer and summer; Table S2, Figure 1). A full description of the climate categories used in this study is provided in Table S2.

To analyse the effects of local temperature and precipitation conditions, we used clutch and egg size records with historical climate variables obtained from the WorldClim bioclimatic database (Fick and Hijmans 2017) at a 10‐min resolution. WorldClim variables represent long‐term climatic conditions (1970–2000), which were used as proxies for typical conditions, to characterise local environmental regimes and are commonly used to describe broad‐scale climatic gradients in ecological studies.

Using the geographic coordinates of each clutch collection site, we extracted the following nine bioclimate variables: isothermality (*T*_iso_), which expresses the ratio between mean diurnal temperature range and annual temperature range; temperature seasonality (*T*_sea_), representing the annual variability in temperature; maximum temperature of warmest month (*T*_max_); minimum temperature of coldest month (*T*_min_); annual mean temperature (*T*_myr_); annual precipitation (*P*_ryr_); precipitation of wettest month (*P*_max_); precipitation of driest month (*P*_min_); and precipitation seasonality (*P*_sea_), which reflects the variability of precipitation throughout the year.

### 2.2 Statistics

We analysed the data using Phylogenetic Generalized Linear Mixed Models (PGLMMs). Since we aimed to observe geo‐climatic variation across the entire *Tyrannus* genus, we set the species taxon as a random variable for clutch and egg size analyses. To control for the phylogenetic effect among related species, we gathered phylogenetic trees of *Tyrannus* species as a subset of the Global Phylogeny of Birds (Jetz et al. 2012). We downloaded 1000 possible phylogenetic trees (Ericson stage 2 backbone) and calculated a single consensus tree summarizing the most common using ‘ape’ (Paradis and Schliep 2019) and ‘phangorn’ (Schliep 2011) packages. Exclusively for egg size, and recognising intraclutch dependence (Christians 2002), we used clutch identity to analyse climate effects on kingbirds' egg size (random slope). The analyses were divided into four geo‐climatic scales: from largest to smallest—latitude, main climate, subclimate and local historical index of temperature and precipitation. For each geo‐climatic scale, a pair of analyses were performed for clutch and egg size as dependent variables. The latitudinal effect on clutch and egg size was evaluated using the absolute value of latitude, from the clutch collection site, as a fixed variable. Then, we assessed the effect of climate conditions on clutch size and egg size of kingbirds in two other steps. The effect of climate was analysed using the main climates as a fixed variable, and then, within each main climate, we analysed the effect of subclimate on clutch size and egg size, considering associated precipitation and temperature conditions.

We evaluated the smaller‐scale climate effect by combining the local bioclimatic indices as fixed variables. To avoid multicollinearity among climate variables, we first estimated the pairwise Pearson's correlation coefficients. Upon detecting a high correlation among a pair of variables (|*r*| > 0.7), we excluded one of those, keeping the variable we considered most ecologically relevant for the model. An initial model was established with all fixed variables. Then, assessing the weight and importance of each variable for the model using Deviance Information Criterion (DIC), we fitted the models by backward deletion, excluding less weighed variables to build the best‐fixed structure.

All climatic continuous variables adopted were standardised with a mean of zero and a standard deviation of one. Finally, we checked for overdispersion for each model, where no overdispersion was detected. All these steps, from the construction of the dataset to the analyses, were performed using the software R (R Core Team 2025) where we used the ‘MCMCglmm’ package (Hadfield 2010) to analyse the PGLMM.

## 3 Results

The initial compilation resulted in 2931 clutches and 9529 eggs for all 13 kingbird species. Photographs were obtained for 1657 clutches from egg collections. After taxonomic verification and checks to ensure that clutch sizes were within the ranges reported for each species in the literature, as well as consistency in geographic distribution, the dataset was reduced to 1358 clutches and 4750 eggs collected over a period of 158 years (1858–2016) (Figure S3).

Because five species ( *Tyrannus niveigularis* , *T. crassirostris* , *T. cubensis* , *T. albogularis* and *T. caudifasciatus* ) were represented by very few clutches, we excluded from further analyses, together representing only 36 clutches and 70 eggs. The trimmed dataset therefore comprised 1332 clutches and 4680 measured eggs from eight species ( *Tyrannus melancholicus* , *T. savana* , *T. dominicensis* , *T. tyrannus* , *T. verticalis* , *T. couchii* , *T. vociferans* and *T. forficatus* ) (Table S1; Figure S4). These records covered the full latitudinal extent of the genus across the Americas and included four major climates and 17 subclimates (Table S2). The overlap between breeding records and local temperature and precipitation variables resulted in a final analytical dataset of 1139 clutches and 4069 eggs used in the statistical analysis.

**FIGURE 2:**
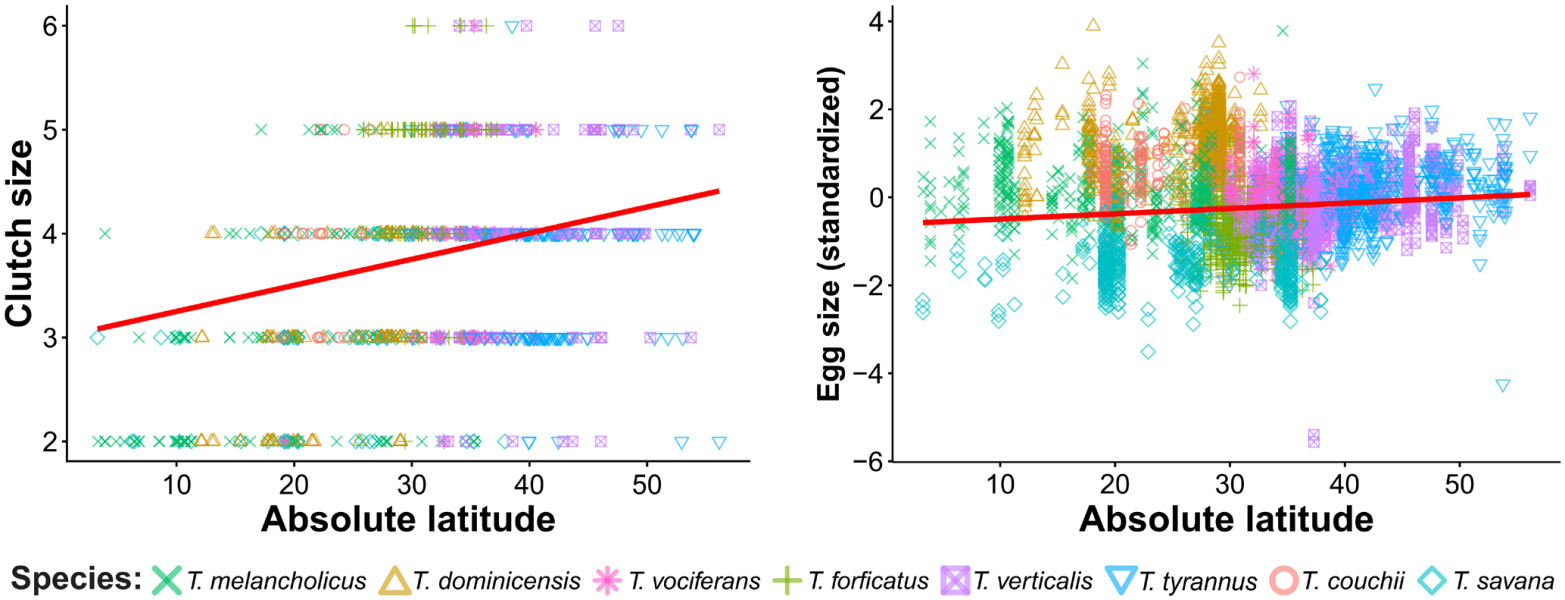
Relationships between clutch size (A) and egg size (B) of kingbirds and absolute latitude by Phylogenetic Generalized Linear Mixed Models (PGLMMs).

**FIGURE 3:**
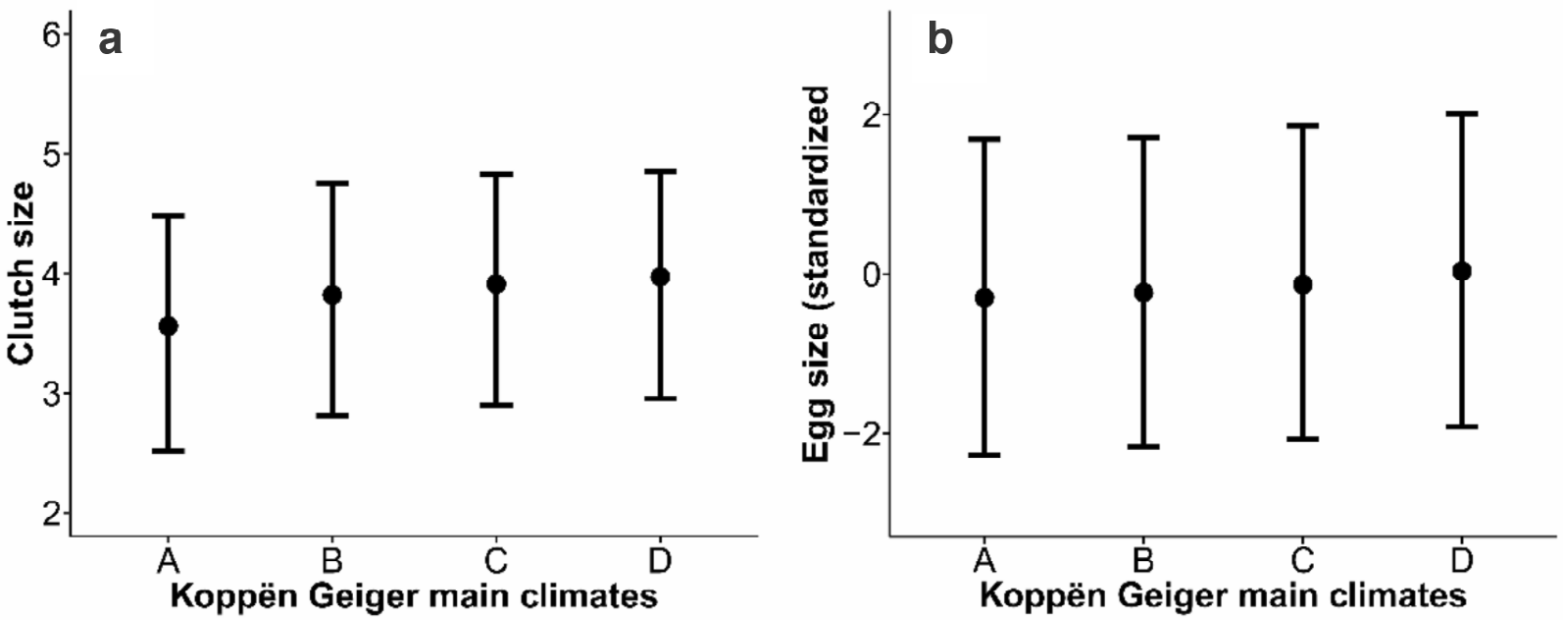
Variation in the clutch size (a) and egg size (b) of kingbird species in equatorial (A), arid (B), warm temperate (C) and snow (D) climates classified from (Kottek et al. 2006). Egg size variation was standardised with a mean of zero and a standard deviation of one.

The PGLMM revealed that clutch size increased significantly with latitude (*β* = 0.02 ± 0.003, Table 1, Figure 2A). Clutch size of kingbirds also varied among the main climates (Table 2, Figure 3a). Taking the equatorial climates as the intercept, where the clutches were smaller, clutch sizes were larger in all other climates. Among all the main climates, the snow climate had the largest clutches, followed by warm temperate and arid climates. Kingbirds' egg size also increased with latitude (*β* = 0.01 ± 0.002, Table 1, Figure 2B). Like clutch size, egg size was smaller in equatorial climates and larger in snow climates, followed by warm temperate climates (Table 2, Figure 3b). However, there was no difference in egg size between equatorial and arid climates.

**TABLE 1:** A summary of the Phylogenetic Generalized Linear Mixed Models investigating latitudinal variation in clutch and egg sizes of kingbirds.

Predictors	Clutch size			Egg size		
	Estimates	SE	CI	Estimates	SE	CI
Intercept	2.99**	0.54	−1.92 to 4.11	−0.61	1.01	−2.61 to 1.51
Absolute latitude	0.02***	0.003	0.01 to 0.03	0.01***	0.002	0.00 to 0.01

*Note:* Egg size was standardised with a mean of zero and a standard deviation of one. Significant effects are classified by *p*‐value.

* *p* < 0.05.

** *p* < 0.01.

*** *p* < 0.001.

**TABLE 2:** A summary of the Phylogenetic Generalized Linear Mixed Models investigating variation in clutch and egg sizes of kingbirds as a function of the main Koppën‐Geiger main climates.

Predictors	Clutch size			Egg size		
	Estimates	SE	CI	Estimates	SE	CI
(A) Equatorial climates (Intercept)	3.56***	0.49	2.52 to 4.48	−0.30	0.97	−2.27 to 1.68
(B) Arid climates	0.26***	0.08	0.11 to 0.42	0.06	0.03	−0.02 to 0.14
(C) Warm Temperate climates	0.35***	0.06	0.23 to 0.47	0.17***	0.03	0.10 to 0.23
(D) Snow climates	0.41**	0.11	0.18 to 0.63	0.33***	0.05	0.23 to 0.44

*Note:* Egg size was standardised with a mean of zero and a standard deviation of one. Significant effects are classified by *p*‐value.

* *p* < 0.05.

** *p* < 0.01.

*** *p* < 0.001.

**FIGURE 4:**
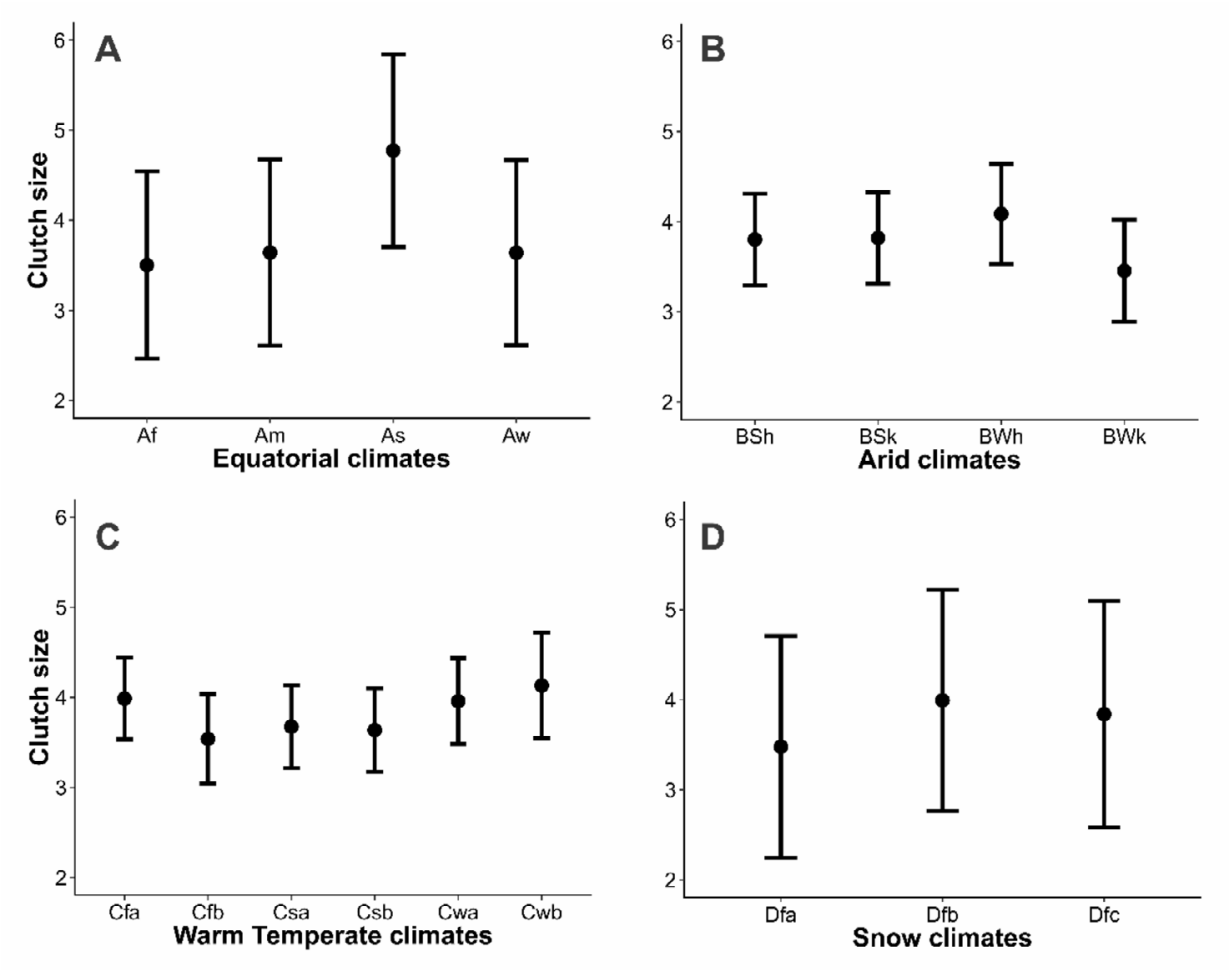
Variation in the clutch size of kingbird species in subclimates of equatorial (A), arid (B), warm temperate (C) and snow (D) climates, classified by (Kottek et al. 2006).

**FIGURE 5:**
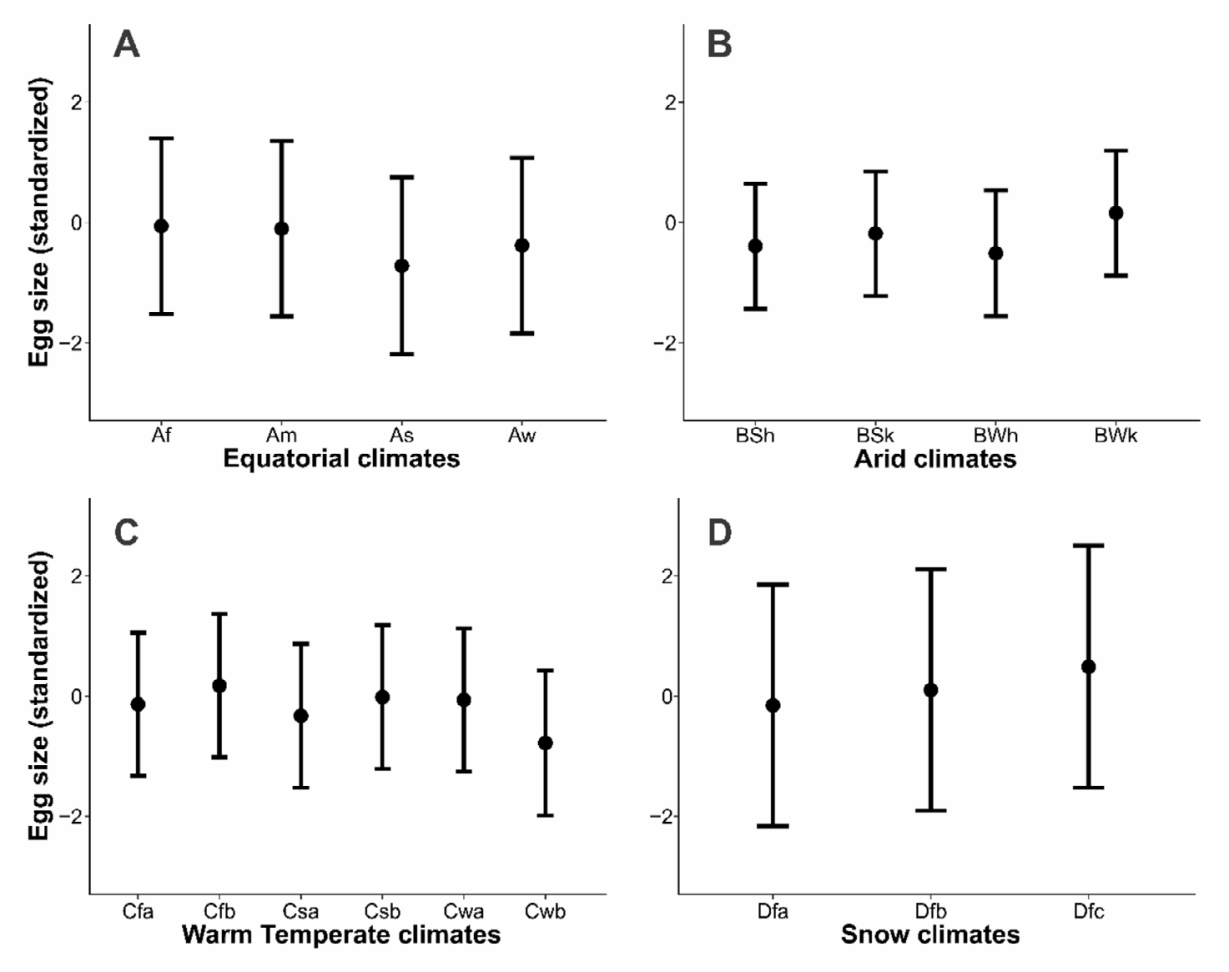
Variation in the egg size of kingbird species in subclimates of equatorial (A), arid (B), warm temperate (C) and snow (D) climates, classified by (Kottek et al. 2006). Egg size variation was standardised with a mean of zero and a standard deviation of one.

There was a significant variation in clutch size among equatorial subclimates (Table 3). Clutches were larger (Figure 4A), and eggs were smaller (Figure 5A) in the *As* subclimate, that is characterised by dry summers with minimum precipitation of < 60 mm. Eggs were also slightly smaller in the *Aw* subclimate than the *Af* subclimate (Figure 5A). Only egg size showed significant variation among arid subclimates (Table 3). Kingbird eggs were larger in the *BSk* and even larger in the *BWk* subclimate (Figure 5B). In warm temperate subclimates, clutch size was significantly smaller in *Cfb* and *Csb* climates (Table 3, Figure 4C). Egg sizes were larger in the *Cfb* but smaller in the *Cwb*, characterised by dry winters and warm summers, followed by *Csa* subclimate. In snow climates, the *Dfb* climate showed the largest clutches (Table 3, Figure 4D), and eggs were significantly larger in *Dfb* and *Dfc* climates (Figure 5D).

**TABLE 3:** A summary of the Phylogenetic Generalized Linear Mixed Models investigating variation in clutch and egg sizes of kingbirds as a function of the Köppen‐Geiger subclimates.

Predictors	Clutch size			Egg size		
	Estimates	SE	CI	Estimates	SE	CI
**Equatorial climates**						
Af (Intercept)	3.50**	1.04	1.64 to 5.73	−0.06	1.46	−2.58 to 2.69
Am	0.14	0.16	−0.18 to 0.45	−0.04	0.09	−0.23 to 0.15
As	1.27***	0.31	0.67 to 1.87	−0.66***	0.15	−0.97 to −0.36
Aw	0.14	0.14	−0.13 to 0.43	−0.32***	0.08	−0.50 to −0.15
**Arid climates**						
BSh (Intercept)	3.80***	0.50	2.87 to 4.82	−0.39	1.03	−2.18 to 1.69
BSk	0.02	0.12	−0.22 to 0.26	0.21***	0.04	0.13 to 0.30
BWh	0.28	0.25	−0.21 to 0.80	−0.12	0.09	−0.29 to 0.06
BWk	−0.35	0.29	−0.91 to 0.23	0.55***	0.10	0.34 to 0.78
**Warm temperate climates**						
Cfa (Intercept)	3.99***	0.45	3.06 to 5.00	−0.14	1.19	−2.48 to 2.00
Cfb	−0.45*	0.22	−0.90 to −0.01	0.31**	0.11	0.10 to 0.53
Csa	−0.31	0.16	−0.63 to 0.01	−0.19**	0.07	−0.34 to −0.04
Csb	−0.35*	0.16	−0.67 to −0.03	0.12	0.07	−0.03 to 0.26
Cwa	−0.03	0.14	−0.29 to 0.26	0.07	0.07	−0.07 to 0.22
Cwb	0.15	0.39	−0.56 to 0.93	−0.64***	0.20	−1.04 to −0.25
**Snow climates**						
Dfa (Intercept)	3.48*	1.23	2.82 to 4.33	−0.15	2.10	−1.93 to 1.22
Dfb	0.52*	0.20	0.12 to 0.91	0.25**	0.08	0.08 to 0.42
Dfc	0.36	0.35	−0.29 to 1.10	0.64***	0.16	0.32 to 0.96

*Note:* Egg size was standardised with a mean of zero and a standard deviation of one. Significant effects are classified by *p*‐value.

* *p* < 0.05.

** *p* < 0.01.

*** *p* < 0.001.

**FIGURE 6:**
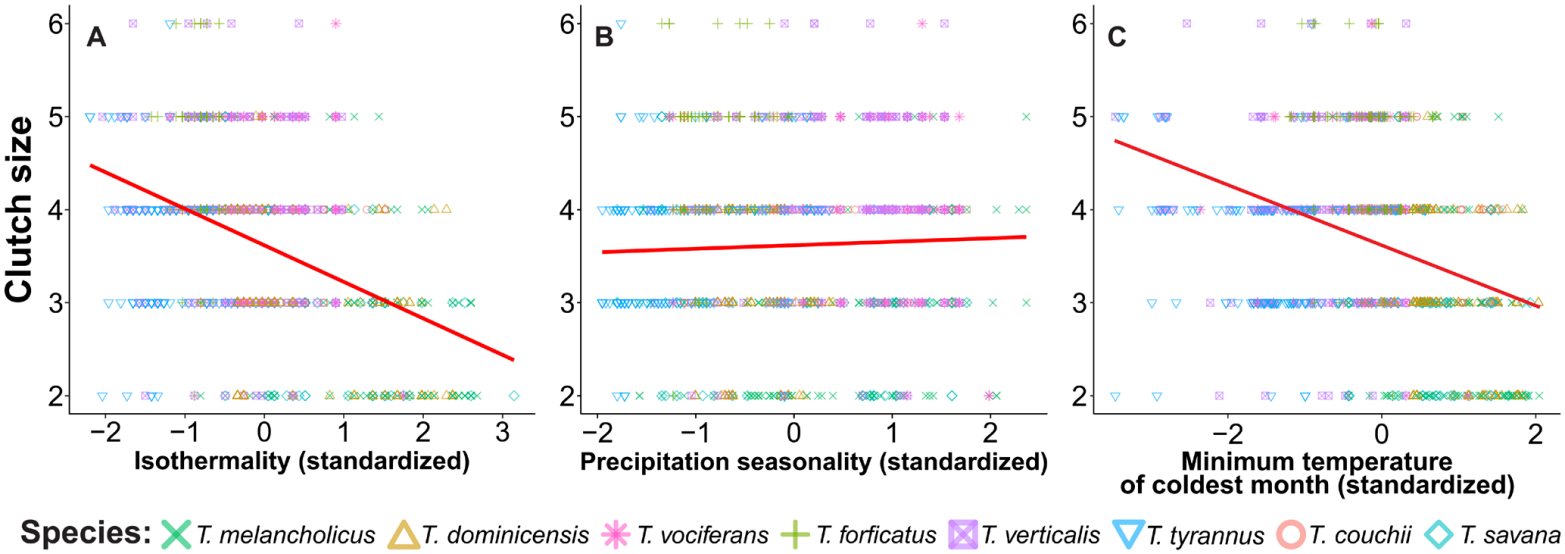
Relationships between clutch size of kingbirds and climatic parameters by Phylogenetic Generalized Linear Mixed Models (PGLMM). The backward selection resulted in the model with the variables: (A) isothermality (*T*_iso_), (B) precipitation seasonality (*P*_sea_) and (C) minimum temperature of the coldest month (*T*_min_).

The best‐adjusted PGLMM for clutch size was composed of three climatic variables (Table 4), including isothermality (*β* = −0.40 ± 0.04; Figure 6A), seasonality of precipitation (*β* = 0.17 ± 0.02; Figure 6B), and minimum temperature of the coldest month (*β* = 0.11 ± 0.04; Figure 6C). In this model, all variables had significant coefficients. This model indicated that kingbirds' clutch size is larger in locations with less stable annual temperatures, higher seasonal precipitation, and the coldest month of the year with higher minimum temperatures.

**TABLE 4:** Phylogenetic generalized linear mixed models, adjusted by the backward selection, for clutch and egg sizes and long‐term mean parameters of precipitation and temperature.

Predictors	Clutch size			Egg size		
	Estimates	SE	CI	Estimates	SE	CI
Intercept	3.79***	0.38	3.10 to 4.61	−0.19	0.96	−2.13 to 1.58
Isot	−0.40***	0.04	−0.49 to −0.32	—	—	—
*P*_min_	—	—	—	0.08***	0.01	0.05 to 0.10
*P*_sea_	0.17***	0.02	0.11 to 0.22	—	—	—
*T*_max_	—	—	—	−0.08***	0.01	−0.10 to −0.06
*T*_min_	0.11*	0.04	0.03 to 0.19	−0.11***	0.01	−0.13 to −0.07

*Note:* Significant effects are classified by *p*‐value. Continuous variables were standardised with a mean of zero and a standard deviation of one.

* *p* < 0.05.

** *p* < 0.01.

*** *p* < 0.001.

**FIGURE 7:**
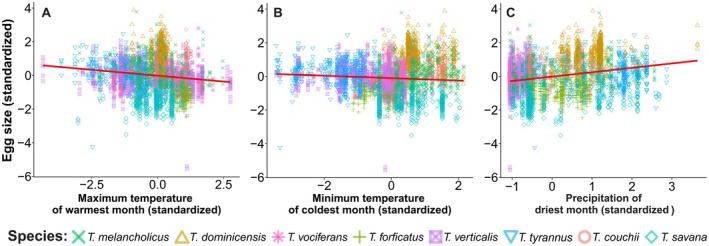
Relationships between egg size of kingbirds and climatic parameters by Phylogenetic Generalized Linear Mixed Models (PGLMMs). The backward selection resulted in the model with the variables: (A) maximum temperature of the warmest month (*T*_max_), (B) minimum temperature of the coldest month (*T*_min_) and (C) precipitation of the driest month (*P*_min_).

For egg size, the best‐adjusted PGLMM had three variables (Table 4), including precipitation of the driest month (*β* = 0.08 ± 0.01; Figure 7A), maximum temperature of the warmest month (*β* = −0.08 ± 0.01; Figure 7B), and minimum temperature of the coldest month (*β* = −0.11 ± 0.01; Figure 7C). All three variables that compound the model were significant. These model coefficients show that kingbirds' egg sizes are larger where the year's driest month has more rainfall and tend to be smaller where the warmest month has higher maximum temperatures and the coldest month has higher minimum temperatures.

## 4 Discussion

Kingbirds' clutches were larger at sites with higher rainfall seasonality (*P*_sea_) and thermal variation (*T*_iso_). Species inhabiting more seasonal environments tend to have larger clutches, especially related to temperature variation (Jetz et al. 2008), a pattern consistent with classic life‐history theory. The increase in seasonality with latitude is one of the most accepted explanations for bird clutch size (Ashmole 1963; Lundblad and Conway 2021). Ashmole's hypothesis proposes that harsher winter conditions increase mortality, primarily through food limitation, thereby reducing population density prior to breeding season. In resident populations, harsh conditions may also exacerbate the regulatory constraints, potentially increasing vulnerability during winter. However, the temperature variability parameter relevant for clutch size was not temperature seasonality (*T*_sea_) but isothermality (*T*_iso_). While temperature seasonality is based on average temperatures, isothermality uses monthly and annual ranges, with maximum and minimum temperatures (O'Donnell and Ignizio 2012). Extreme temperatures have been a better predictor than average temperatures (Schaper et al. 2012) because temperature parameters have a higher potential to cause physiological stress. Our results also show that clutches tend to be larger in environments with higher precipitation seasonality, corroborating Ashmole's hypothesis. Additionally, in Equatorial climate, kingbirds tend to have larger clutches in subclimates with dry summers (*As*).

With respect to precipitation, support for our predictions depended on both the response variable and the climatic metric considered. For clutch size, we found support for prediction (b), as clutch size was associated with precipitation seasonality, but not for prediction (d), since absolute precipitation was not retained in the best‐supported models. This suggests that temporal variation in resource availability, rather than total rainfall, is more relevant for explaining clutch size. Precipitation strongly influences invertebrate populations (Pinheiro et al. 2002), which are key for egg production in insectivorous birds such as *Tyrannus* (Fitzpatrick 1980; Murphy 1983; Blancher and Robertson 1987; McWilliams et al. 2016), and lower seasonality may keep insect populations at equilibrium throughout the year (Wolda 1988). In contrast, egg size showed partial support for prediction (d), as eggs were larger at sites with higher precipitation during the driest months of the year (*P*_min_). This suggests that conditions outside the breeding season may influence maternal condition prior to reproduction, potentially affecting egg size, consistent with lagged climatic effects on life‐history traits (e.g., O'Neil et al. 2025). Because larger eggs tend to produce larger and potentially higher‐fitness offspring (Krist 2011), more benign breeding seasons would allow the production of higher‐fitness offspring, although egg quality is also influenced by composition, and not only size (Birchard and Deeming 2015).

Sites with warmer winters (*T*_min_ >), were associated with larger clutches and smaller eggs. Similarly, subclimates characterised by colder winter temperatures showed reduced clutches (Csb), reinforcing the role of winter conditions in shaping reproductive investment. In contrast, egg size generally decreased with increasing temperature, as sites with higher maximum temperatures in the warmest month (*T*_max_) were associated with smaller eggs. However, subclimate analyses suggest that this relationship depends on environmental context. Egg size was largest in cooler arid subclimates (BSk, BWk), indicating that the negative effect of temperature on egg size may be stronger in warm, arid environments than in warm, humid ones. This pattern likely reflects an interaction between thermal constraints and resource availability. In arid regions, high temperatures may increase physiological stress, such as dehydration, mainly in water‐limited environments (Sauve et al. 2021; Schifferli et al. 2014; Whitfield et al. 2015). In contrast, warm and humid environments tend to support higher primary productivity and insect availability (Grüebler et al. 2008), which may buffer the negative effects of temperature on reproductive investment. Together, these results suggest that egg size is shaped by a balance between thermal stress and resource availability across environmental gradients.

In Snow climates, *Tyrannus* only breeds in wet subclimates (Df*), but larger clutches occur only in subclimates that have warm summers (*Dfb*), while larger eggs are found in subclimates that have cool summers (*Dfc*). The high precipitation in this last subclimate, combined with the increased egg size in environments with more rain in the driest month of the year (*P*_min_ >), also shows the importance of rainfall for kingbird's egg size, even though one study has found no significant effect of precipitation for one kingbird species (Murphy 1983). Even if precipitation occurs during winter, climatic events tend to influence individuals and populations in subsequent seasons, supporting the importance of the time lag effect (Marra et al. 2015). Kingbirds' clutch sizes tended to be larger in snow climates, followed by warm temperate and arid climates, and were smaller in equatorial climates, though with confidence interval overlap and lack of difference among some main climates. This progression does not coincide with summer temperatures or precipitation but coincides with a decrease in winter temperatures. Higher latitude species tend to cover a higher latitudinal range and be better tolerant to temperature variation (Stevens 1989). In addition, local and regional sites with lower minimum winter temperatures tend to have smaller clutches. The progression of egg sizes followed the trend observed for clutch size, and snow climates had the largest eggs. Particularly for egg size, equatorial and arid climates did not differ. Our findings so far indicate a combination of local factors that underlie the geographic variation in kingbird breeding traits.

The thermal conditions of colder environments may impose greater energetic demands on female kingbirds, which perform uniparental incubation (Blancher and Robertson 1985; Murphy 1996), increasing the difficulty of maintaining optimal incubation temperatures (Gillette et al. 2021). Larger eggs may help mitigate these constraints, as they lose heat more slowly and can buffer temperature fluctuations, potentially reducing the frequency or cost of rewarming during incubation recesses (Krist 2011; Gillette et al. 2021). At the same time, environmental context may modulate this pattern. Warmer environments, particularly when arid, may impose resource limitations that constrain egg production, whereas warm and humid environments tend to support higher primary productivity and resource availability. Thus, variation in egg size likely reflects a balance between thermal constraints and resource availability, with larger eggs favoured in colder environments due to their thermoregulatory advantages, and smaller eggs occurring in warmer, resource‐limited conditions (Järvinen and Ylimaunu 1986; Gebhardt‐Henrich and Richner 1998).

The latitudinal variation in kingbird's clutch size corroborates again the pattern observed since Moreau (1944), increasing significantly toward the poles (Cody 1966). Environments with more seasonal climates and temperature variations have larger clutches (Griebeler et al. 2004), and climate seasonality was a common factor among the climates and climatic conditions that allowed kingbirds to increase their clutches at higher latitudes. However, we found that harsher winter conditions alone did not promote larger clutches. This result supports the idea that seasonality, rather than winter severity per se, drives variation in clutch size, as proposed by Ricklefs (1980). Thus, although harsh winters may reduce population density and contribute to an increase in per capita resource availability during the breeding season (Lv et al. 2023), it is the seasonal contrast between periods of low and high productivity that determines the surplus resources available for reproduction. Egg size followed the same trend as clutch size and increased with latitude. As an important factor for primary productivity, regions with milder winters can reflect a reproductive season with greater resource abundance, but also high competitiveness (Newton 1998). The progression of egg size with latitude appears to be negatively related to temperatures, as observed for other vertebrate groups (Sheader 1996; Feiner et al. 2016). Higher latitudes share the climatic characteristics that enable kingbirds to lay larger eggs.

As the explanation of life history patterns is based on complex interactions of traits (Bennett and Owens 2002) and trade‐offs based on resource allocation (Stearns 1992), investment in clutch size can be associated with adjustments in egg size (Roff and Fairbairn 2007; Stearns 1992). This adjustment, however, depends on the short‐term strategies females adopt under environmental conditions in the reproduction cycle (Aranzamendi et al. 2019). In our dataset, *Tyrannus* species did not show a consistent trade‐off between clutch and egg size, with relationships varying among species (positive, negative or absent). This indicates that trade‐offs in these traits may be species‐specific within the genus rather than a general pattern, which is consistent with previous studies reporting weak or absent correlations between clutch and egg size in birds (Christians 2002; Sakai 2021).

In addition to the direct parental effect on clutch and egg size, much of the variation of an offspring trait is due to environmental quality. *Tyrannus tyrannus* , for example, changes its reproductive performance interannually as a function of environmental quality (Blancher and Robertson 1985). Since species adapt their reproductive traits to a given climate, interannual variation in climatic conditions tends to influence the plasticity of their traits (Visser 2008). Kingbirds have breeding characteristics that are dependent on climatic conditions. Members of this genus have high fidelity to breeding sites (Blancher and Robertson 1985; Murphy 1996) and select habitats based on climatic parameters, as is the case for *T. savana* and *T. tyrannus* , where temperature is a cue for breeding sites (MacPherson et al. 2018) and *T. savana* uses precipitation to select wintering sites (Jahn et al. 2013). In addition, heritability reinforces the relationship between species and local climate across generations (Christians 2002). Thus, reproductive traits are shaped by the interaction between environmental constraints and adaptive responses, which may vary across climatic contexts. It is essential to understand how climates influence reproductive traits and how species respond to variation in environmental conditions. Future studies incorporating explicit interactions among climatic variables at finer temporal and spatial scales may help disentangle the joint effects of temperature and water availability on reproductive traits.

Climate unpredictability is one of the most significant factors experienced by species through global climate change (Hansen et al. 2012), with negative effects on their fitness (McNamara et al. 2011). Kingbirds and other species are more responsive to extreme than average temperatures (Schaper et al. 2012). Global climate change will most impact extreme climate parameters (Marcelino et al. 2020). Heat waves, droughts and excessive rainfall are consequences of climate change and can affect birds depending on which stage of reproduction they experience these conditions (Cady et al. 2019; Sauve et al. 2021). Projections of future climate change scenarios show that birds are more vulnerable to future thermal stresses, even more than mammals (Riddell et al. 2021).

In conclusion, our findings show that kingbird clutch and egg sizes vary according to regional and local climatic conditions, and that these relationships help explain the latitudinal cline of reproductive investment. However, it remains unclear whether this variation reflects adaptive life‐history strategy or a physiological response to environmental conditions. Further research linking climatic conditions to life‐history traits is therefore essential. Importantly, species from different biogeographic regions may exhibit shared or divergent responses to climatic conditions, and responses observed in Nearctic species may not necessarily apply to Neotropical taxa. Furthermore, long‐term and large‐scale datasets are essential to understand how global climate change will affect species and how their plasticity may buffer future environmental changes.

## Author Contributions

**Marcelo Assis:** conceptualization (lead), data curation (lead), formal analysis (lead), funding acquisition (lead), investigation (lead), methodology (lead), project administration (supporting), supervision (supporting), validation (lead), visualization (lead), writing – original draft (lead), writing – review and editing (equal). **Neander M. Heming:** data curation (supporting), methodology (supporting), writing – review and editing (supporting). **Miguel Â. Marini:** conceptualization (supporting), data curation (lead), funding acquisition (lead), investigation (supporting), project administration (lead), writing – original draft (supporting), writing – review and editing (supporting).

## Funding

M.A. held fellowship from Coordenação de Aperfeiçoamento de Pessoal de Nível Superior (CAPES) and Conselho Nacional de Desenvolvimento Científico e Tecnológico (CNPq) during his doctoral studies. Research funding was provided by Conselho Nacional de Desenvolvimento Científico e Tecnológico (# 473281/2013‐9), Fundação de Apoio à Pesquisa do Distrito Federal (# 0193.000839/2015, # 0193.001090/2016, # 00193.00000962/2018‐40, # 00193‐00001412/2019‐29), and American Museum of Natural History (AMNH). M.Â.M. held a researcher fellowships from Conselho Nacional de Desenvolvimento Científico e Tecnológico (# 305458/2014‐0; # 311081/2019‐3; # 311626/2025‐4). N.M.H. received a postdoc fellowship from Coordenação de Aperfeiçoamento de Pessoal de Nível Superior. We thank DPI/BCE for publishing support to M.A.M.: “EDITAL DPI/BCE/UNB # 001/2026”.

## Ethics Statement

The authors have nothing to report.

## Consent

The authors have nothing to report.

## Conflicts of Interest

The authors declare no conflicts of interest.

## Supporting information


**Figure S1:** Process of obtaining photographic records of bird clutches in egg collections. (A) Smithsonian National Museum of Natural History (Washington, DC); (B) American Museum of Natural History (New York); (C) Harvard Museum of Comparative Zoology (Cambridge); (D) Museo Argentino de Ciencias Naturales (Buenos Aires); and (E) Western Foundation of Vertebrate Zoology (Camarillo).
**Figure S2:** Digital extraction process of the egg measurements using ImageJ software, following Troscianko's (2014) methodology: (1) Defining points at the main extremities; (2) Viewing and readjusting the generated egg circumference; (3) Setting the scale; and (4) Calculated dimensions.
**Figure S3:** Distribution of breeding records gathered from 13 species of the *Tyrannus* genus ( *T. albogularis* ; *T. caudifasciatus* ; *T. couchii* ; *T. crassirostris* ; *T. cubensis* ; *T. dominicensis* ; *T. forficatus* ; *T. melancholicus* ; *T. niveigularis* ; *T. savana* ; *T. tyrannus* ; *T. verticalis* ; and *T. vociferans* ). With different colours and shapes by species, dots show the locations where the clutches were collected.
**Figure S4:** Distribution of breeding records gathered from eight analysed species of the *Tyrannus* genus ( *T. couchii* ; *T. dominicensis* ; *T. forficatus* ; *T. melancholicus* ; *T. savana* ; *T. tyrannus* ; *T. verticalis* ; and *T. vociferans* ). With different colours and shapes by species, dots show the locations where the clutches were collected.
**Table S1:** Dataset of clutches and eggs of 13 kingbird species, photographed in 23 egg collections in South America, the USA and Europe. The number outside the parentheses refers to the clutches, while the value within the parentheses refers to the number of eggs.
**Table S2:** Climates according to Köppen‐Geiger and their temperature and precipitation descriptions for which the respective *Tyrannus* species were recorded. The sample size of clutches and eggs is shown for each main climate and sub‐climate for each species—*Source:* Adapted from Kottek et al. (2006).

## Data Availability

Data available from the Dryad digital repository: https://doi.org/10.5061/dryad.qz612jmmd.
