# Supplementary material for: Multiple Spatial and Climatic Conditions Affect Kingbird Flycatchers' Clutch and Egg Sizes
Source: Ecol Evol. 2026 Apr 19;16(4):e73528. doi: 10.1002/ece3.73528 (PMC13092806; doi:10.1002/ece3.73528)
Supplement: Supplementary file 1 — Figure S1: Process of obtaining photographic records of bird clutches in egg collections. (A) Smithsonian National Museum of Natural History (Washington, DC); (B) American Museum of Natural History (New York); (C) Harvard Museum of Comparative Zoology (Cambridge); (D) Museo Argentino de Ciencias Naturales (Buenos Aires); and (E) Western Foundation of Vertebrate Zoology (Camarillo). Figure S2: Digital extraction process of the egg measurements using ImageJ software, following Troscianko's (2014) methodology: (1) Defining points at the main extremities; (2) Viewing and readjusting the generated egg circumference; (3) Setting the scale; and (4) Calculated dimensions. Figure S3: Distribution of breeding records gathered from 13 species of the Tyrannus genus ( T. albogularis ; T. caudifasciatus ; T. couchii ; T. crassirostris ; T. cubensis ; T. dominicensis ; T. forficatus ; T. melancholicus ; T. niveigularis ; T. savana ; T. tyrannus ; T. verticalis ; and T. vociferans ). With different colours and shapes by species, dots show the locations where the clutches were collected. Figure S4: Distribution of breeding records gathered from eight analysed species of the Tyrannus genus ( T. couchii ; T. dominicensis ; T. forficatus ; T. melancholicus ; T. savana ; T. tyrannus ; T. verticalis ; and T. vociferans ). With different colours and shapes by species, dots show the locations where the clutches were collected. Table S1: Dataset of clutches and eggs of 13 kingbird species, photographed in 23 egg collections in South America, the USA and Europe. The number outside the parentheses refers to the clutches, while the value within the parentheses refers to the number of eggs. Table S2: Climates according to Köppen‐Geiger and their temperature and precipitation descriptions for which the respective Tyrannus species were recorded. The sample size of clutches and eggs is shown for each main climate and sub‐climate for each species—Source: Adapted from Kottek et al. (2006). [file ECE3-16-e73528-s001.docx]

# SUPPORTING INFORMATION

Table S1 – Dataset of clutches and eggs of 13 kingbird species, photographed in 23 egg collections in South America, the USA and Europe. The number outside the parentheses refers to the clutches, while the value within the parentheses refers to the number of eggs.

|  | *Tyrannus* *verticalis* | *Tyrannus* *dominicensis* | *Tyrannus* *vociferans* | *Tyrannus* *melancholicus* | *Tyrannus* *forficatus* | *Tyrannus* *tyrannus* | *Tyrannus* *couchii* | *Tyrannus* *savana* | *Tyrannus* *albogularis* | *Tyrannus* *caudifasciatus* | *Tyrannus* *niveigularis* | *Tyrannus* *crassirostris* | *Tyrannus* *cubensis* | Total by collection |
| --- | --- | --- | --- | --- | --- | --- | --- | --- | --- | --- | --- | --- | --- | --- |
| AMNH - New York | 12 (41) | 34 (109) | 5 (21) | 4 (11) |  |  |  | 3 (10) |  |  |  |  |  | 58 (192) |
| CAS - San Francisco |  |  |  | 1 (4) |  |  |  | 1 (3) |  |  |  |  |  | 2 (7) |
| COMB - Brasília |  |  |  | 17 (28) |  |  |  | 13 (25) | 2 (4) |  |  |  |  | 32 (57) |
| DMNH - Delaware | 20 (78) | 46 (138) |  | 23 (64) |  |  |  | 27 (86) |  |  |  |  |  | 116 (366) |
| IAvH - Villa de Leyva | 3 (9) | 1 (4) | 1 (5) | 2 (4) | 1 (5) | 7 (28) |  | 1 (3) |  |  |  |  |  | 16 (58) |
| MACN - Buenos Aires |  |  |  | 2 (6) |  | 1 (4) |  | 8 (25) |  |  |  |  |  | 11 (35) |
| MCZ - Harvard | 12 (49) | 7 (20) | 1 (4) | 1 (2) |  | 8 (27) |  | 2 (8) |  | 1 (2) |  |  |  | 32 (112) |
| MLP - La Plata |  |  |  | 1 (2) |  |  |  | 3 (9) |  |  |  |  |  | 4 (11) |
| MLUH - Halle |  | 4 (8) |  | 8 (22) |  |  |  | 4 (10) |  |  | 1 (1) |  |  | 17 (41) |
| MN - Rio de Janeiro |  |  |  | 8 (23) | 1 (5) | 1 (3) |  | 12 (36) |  |  |  |  |  | 22 (67) |
| MPEG - Belém |  |  |  | 1 (1) |  |  |  |  |  |  |  |  |  | 1 (1) |
| MVZ - Berkeley |  |  | 1 (1) | 2 (2) |  |  |  | 2 (5) |  |  |  |  |  | 5 (8) |
| MZUSP - São Paulo | 1 (4) |  |  | 4 (14) |  | 1 (3) |  | 1 (3) |  |  |  |  |  | 7 (24) |
| NBCN - Leiden |  | 3 (7) |  | 5 (15) |  |  |  |  |  |  |  |  |  | 8 (22) |
| NHM - Tring | 7 (24) | 7 (14) | 2 (7) | 33 (79) | 4 (16) | 14 (46) |  | 12 (40) |  | 2 (8) | 2 (3) | 1 (2) |  | 84 (239) |
| NMBE - Berna |  |  |  | 6 (16) |  |  |  |  |  |  |  |  |  | 6 (16) |
| NMS - Edinburgh |  | 1 (3) |  | 2 (5) |  |  |  | 6 (16) |  |  |  |  |  | 9 (24) |
| NMW - Viena |  |  | 1 (3) | 1 (4) | 1 (5) | 2 (6) |  | 1 (3) |  |  |  |  |  | 6 (21) |
| SBCM - San Bernardino |  | 1 (2) |  | 20 (53) |  |  | 1 (4) |  |  |  |  |  |  | 22 (59) |
| UFRRJ - Rio de Janeiro |  |  |  | 1 (3) |  |  |  | 1 (3) |  |  |  |  |  | 2 (6) |
| USNM - Washington | 19 (68) | 26 (72) | 16 (53) | 11 (36) | 2 (10) | 4 (15) |  | 4 (12) |  | 3 (9) |  |  |  | 85 (275) |
| WFVZ - Camarillo | 186 (787) | 109 (361) | 90 (366) | 39 (118) | 125 (561) | 147 (532) | 60 (218) | 36 (112) |  |  | 3 (9) | 4 (17) | 1 (2) | 800 (3083) |
| ZMB - Berlin |  | 5 (10) |  | 1 (2) |  |  |  | 1 (1) |  | 3 (7) | 2 (4) | 1 (2) |  | 13 (26) |
| Total by species | 260 (1060) | 244 (748) | 117 (460) | 193 (514) | 134 (602) | 185 (664) | 61 (222) | 138 (410) | 2 (4) | 9 (26) | 8 (17) | 6 (21) | 1 (2) | 1358 (4750) |
|  |  |  |  |  |  |  |  |  |  |  |  |  |  |  |

TABLE S2 – Climates according to Köppen-Geiger and their temperature and precipitation descriptions for which the respective *Tyrannus* species were recorded. The sample size of clutches and eggs is shown for each main climate and sub-climate for each species—source: Adapted from Kottek et al. (2006).

| Main climate | Main climate sample | Sub-climate | Sub-climate sample | Species |
| --- | --- | --- | --- | --- |
| A  Equatorial climates  (Tmin ≥+18 ◦C) | 309 (838) | Af  Rainforest, fully humid  Pmin ≥ 60 mm | 31 (74) | *Tyrannus* *dominicensis* (12[27]), *Tyrannus* *melancholicus* (12[30]), *Tyrannus* *niveigularis* (3[5]), *Tyrannus* *savana* (4[12]) |
|  |  | Am  Monsoon  Pann ≥ 25(100−Pmin) | 48 (129) | *Tyrannus* *caudifasciatus* (4[12]), *Tyrannus* *couchii* (4[14]), *Tyrannus* *cubensis* (1[2]), *Tyrannus* *dominicensis* (8[23]), *Tyrannus* *melancholicus* (29[71]), *Tyrannus* *savana* (2[7]) |
|  |  | As  Savannah, with dry summer  Pmin < 60 mm in summer | 9 (31) | *Tyrannus* *crassirostris* (2[4]), *Tyrannus* *melancholicus* (7[27]) |
|  |  | Aw  Savannah, with dry winter  Pmin < 60 mm in winter | 221 (604) | *Tyrannus* *albogularis* (2[4]), *Tyrannus* *caudifasciatus* (5[14]), *Tyrannus* *couchii* (30[106]), *Tyrannus* *dominicensis* (42[105]), *Tyrannus* *forficatus* (1[5]), *Tyrannus* *melancholicus* (71[181]), *Tyrannus* *niveigularis* (4[9]), *Tyrannus* *savana* (66[180]) |
| B  Arid climates  (Pann < 10 Pth) | 246 (945) | BSh  Steppe climate  Pann > 5 Pth  Hot Tann ≥+18 ◦C | 79 (291) | *Tyrannus* *couchii* (9[35]), *Tyrannus* *crassirostris* (2[8]), *Tyrannus* *melancholicus* (13[33]), *Tyrannus* *savana* (3[8]), *Tyrannus* *verticalis* (32[128]), *Tyrannus* *vociferans* (20[79]) |
|  |  | BSk  Steppe climate Pann > 5 Pth  Cold Tann <+18 ◦C | 144 (568) | *Tyrannus* *crassirostris* (1[5]), *Tyrannus* *forficatus* (5[23]), *Tyrannus* *melancholicus* (4[16]), *Tyrannus* *savana* (2[5]), *Tyrannus* *tyrannus* (10[36]), *Tyrannus* *verticalis* (77[303]), *Tyrannus* *vociferans* (45[180]) |
|  |  | BWh  Desert climate  Pann ≤ 5 Pth  Hot Tann ≥+18 ◦C | 14 (53) | *Tyrannus* *melancholicus* (7[22]), *Tyrannus* *niveigularis* (1[3]), *Tyrannus* *verticalis* (5[24]), *Tyrannus* *vociferans* (1[4]) |
|  |  | BWk  Desert climate  Pann ≤ 5 Pth  Cold Tann <+18 ◦C | 9 (33) | *Tyrannus* *verticalis* (4[16]), *Tyrannus* *vociferans* (5[17]) |
| C  Warm temperate climate  (-3 ◦C < Tmin <+18 ◦C) | 717 (2649) | Cfa  Fully humid  neither Cs nor Cw  Hot summer  Tmax ≥+22 ◦C | 518 (1882) | *Tyrannus* *couchii* (15[56]), *Tyrannus* *dominicensis* (182[593]), *Tyrannus* *forficatus* (128[574]), *Tyrannus* *melancholicus* (34[89]), *Tyrannus* *savana* (44[144]), *Tyrannus* *tyrannus* (100[356]), *Tyrannus* *verticalis* (14[65]), *Tyrannus* *vociferans* (1[5]) |
|  |  | Cfb  Fully humid  neither Cs nor Cw  Warm summer  not (a) and at least 4 Tmon ≥+10 ◦C | 10 (32) | *Tyrannus* *melancholicus* (2[5]), *Tyrannus* *savana* (1[2]), *Tyrannus* *tyrannus* (5[18]), *Tyrannus* *verticalis* (1[3]), *Tyrannus* *vociferans* (1[4]) |
|  |  | Csa  Dry summer  Psmin < Pwmin, Pwmax > 3 Psmin and Psmin < 40 mm  Hot summer  Tmax ≥+22 ◦C | 75 (306) | *Tyrannus* *crassirostris* (1[4]), *Tyrannus* *verticalis* (58[241]), *Tyrannus* *vociferans* (16[61]) |
|  |  | Csb  Dry summer  Psmin < Pwmin, Pwmax > 3 Psmin and Psmin < 40 mm  Warm summer  not (a) and at least 4 Tmon ≥+10 ◦C | 82 (329) | *Tyrannus* *tyrannus* (1[4]), *Tyrannus* *verticalis* (53[215]), *Tyrannus* *vociferans* (28[110]) |
|  |  | Cwa  Dry winter  Pwmin < Psmin and Psmax > 10 Pwmin  Hot summer  Tmax ≥+22 ◦C | 29 (89) | *Tyrannus* *couchii* (3[11]), *Tyrannus* *melancholicus* (12[33]), *Tyrannus* *savana* (14[45]) |
|  |  | Cwb  Dry winter  Pwmin < Psmin and Psmax > 10 Pwmin  Warm summer  not (a) and at least 4 Tmon ≥+10 ◦C | 3 (11) | *Tyrannus* *melancholicus* (2[7]), *Tyrannus* *verticalis* (1[4]) |
| D  Snow climates  (Tmin ≤-3 ◦C) | 84 (311) | Dfa  Fully humid  Neither Ds (Psmin < Pwmin, Pwmax > 3 Psmin and Psmin < 40 mm) nor Dw (Pwmin < Psmin and Psmax > 10 Pwmin)  Hot summer  Tmax ≥+22 ◦C | 21 (71) | *Tyrannus* *tyrannus* (18[62]), *Tyrannus* *verticalis* (3[9]) |
|  |  | Dfb  Fully humid  Neither Ds (Psmin < Pwmin, Pwmax > 3 Psmin and Psmin < 40 mm) nor Dw (Pwmin < Psmin and Psmax > 10 Pwmin)  Warm summer  not (a) and at least 4 Tmon ≥+10 ◦C | 57 (219) | *Tyrannus* *tyrannus* (48[178]), *Tyrannus* *verticalis* (9[41]) |
|  |  | Dfc  Fully humid  Neither Ds (Psmin < Pwmin, Pwmax > 3 Psmin and Psmin < 40 mm) nor Dw (Pwmin < Psmin and Psmax > 10 Pwmin)  Cool summer and cold winter  not (b) and Tmin >−38 ◦C | 6 (21) | *Tyrannus* *tyrannus* (3[10]), *Tyrannus* *verticalis* (3[11]) |


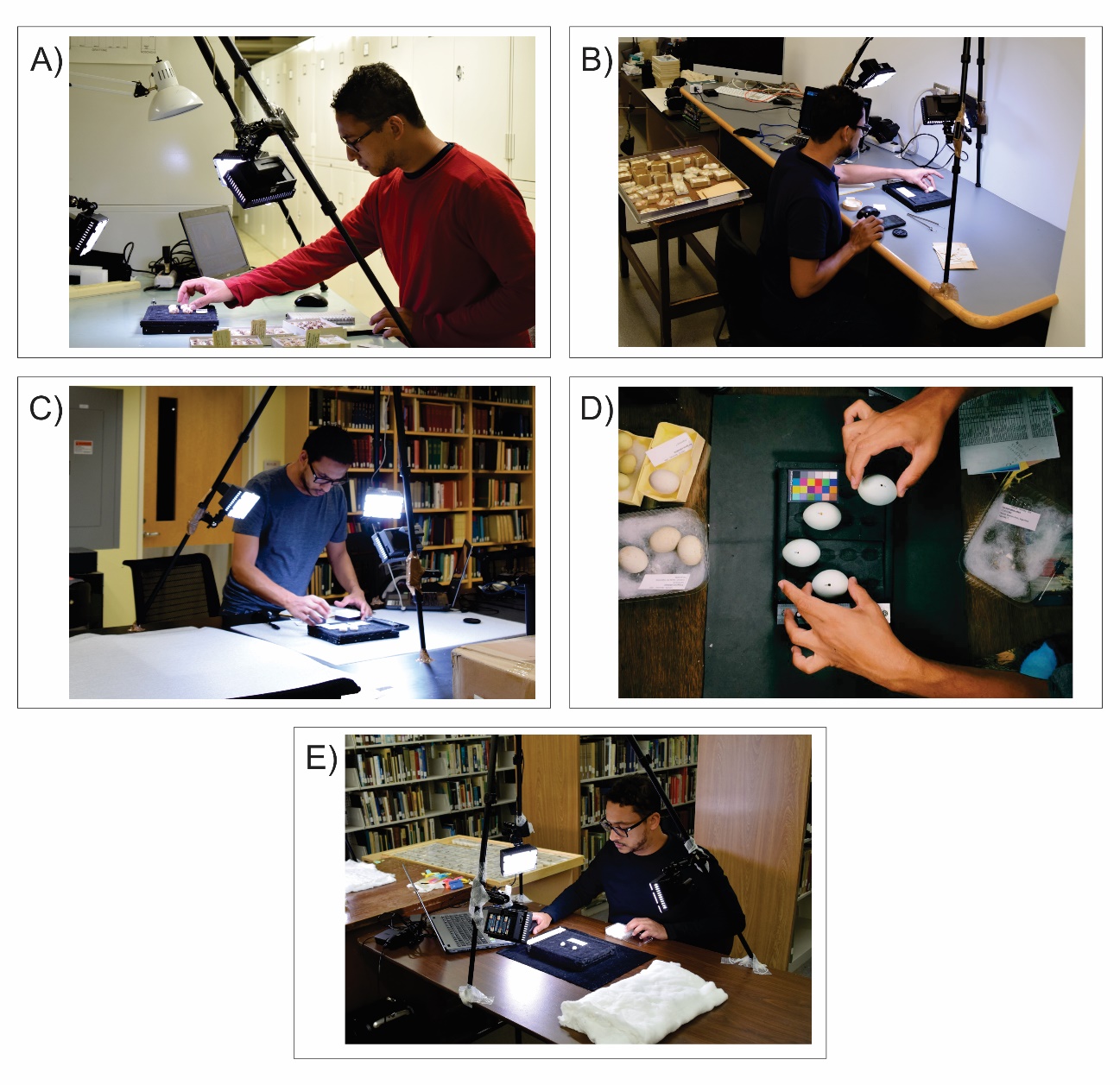


FIGURE S1 - Process of obtaining photographic records of bird clutches in egg collections. A) Smithsonian National Museum of Natural History (Washington, DC); B) American Museum of Natural History (New York); C) Harvard Museum of Comparative Zoology (Cambridge); D) Museo Argentino de Ciencias Naturales (Buenos Aires); and E) Western Foundation of Vertebrate Zoology (Camarillo).


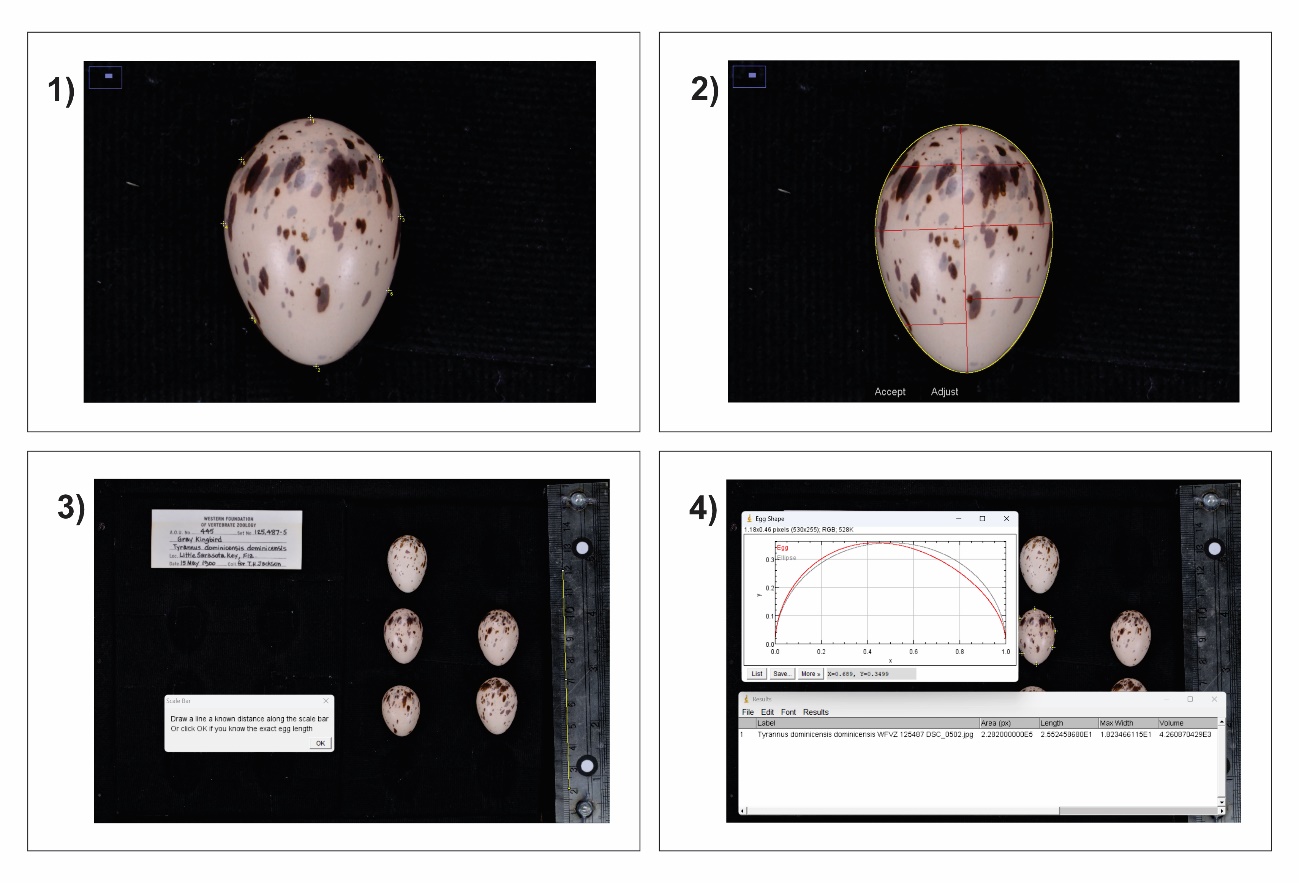


FIGURE S2 – Digital extraction process of the egg measurements using ImageJ software, following Troscianko's (2014) methodology: 1) Defining points at the main extremities; 2) Viewing and readjusting the generated egg circumference; 3) Setting the scale; and 4) Calculated dimensions.


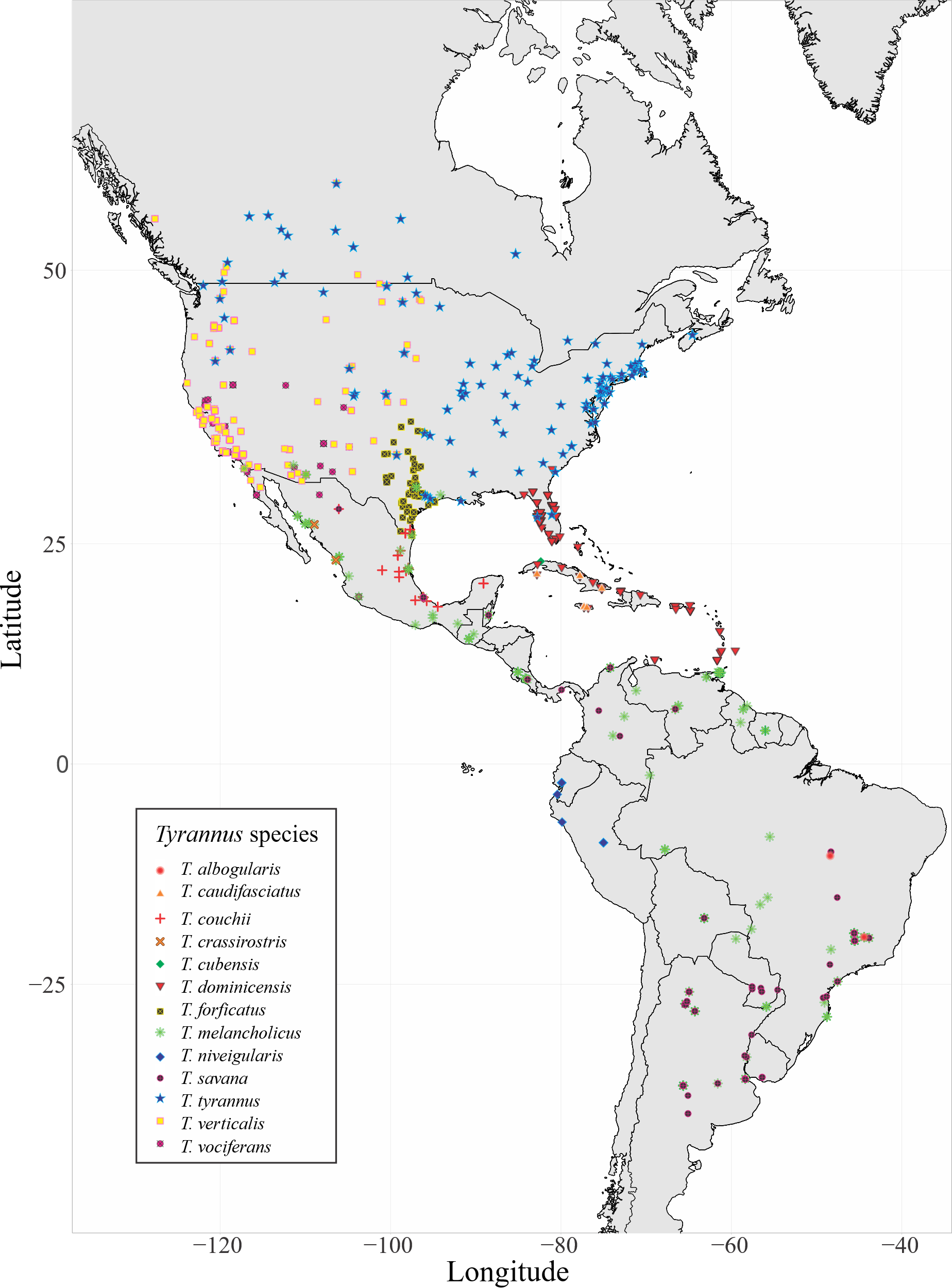


FIGURE S3 - Distribution of breeding records gathered from 13 species of the *Tyrannus* genus *(T. albogularis; T. caudifasciatus; T. couchii; T. crassirostris; T. cubensis; T. dominicensis; T. forficatus; T. melancholicus; T. niveigularis; T. savana; T. tyrannus; T. verticalis*; and *T. vociferans)*. With different colours and shapes by species, dots show the locations where the clutches were collected.


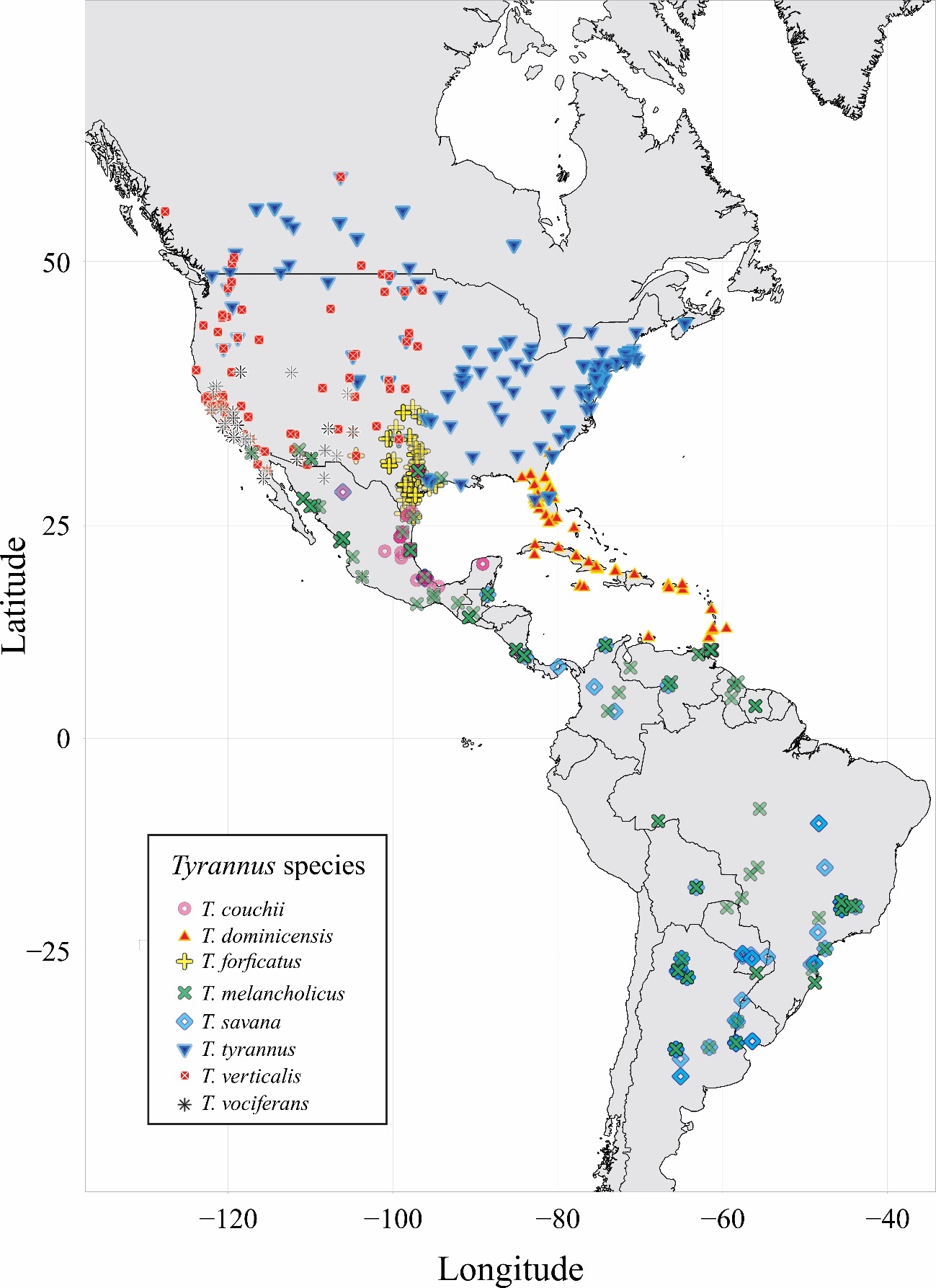


FIGURE S4 - Distribution of breeding records gathered from eight analysed species of the *Tyrannus* genus (*T. couchii; T. dominicensis; T. forficatus; T. melancholicus; T. savana; T. tyrannus; T. verticalis;* and *T. vociferans*). With different colours and shapes by species, dots show the locations where the clutches were collected.
